# The Evolving Role of Extracorporeal In Situ Perfusion Technology in Organ Donor Recovery with Donation After Circulatory Determination of Death Organ Donors

**DOI:** 10.3390/medicina61071276

**Published:** 2025-07-15

**Authors:** Victoria R. Hammond, Marisa E. Franklin, Glen A. Franklin

**Affiliations:** 1 Hiram C Polk Jr MD Department of Surgery, University of Louisville, Louisville, KY 40202, USA; victoria.hammond@louisville.edu; 2 The Mahurin Honors College, Western Kentucky University, Bowling Green, KY 42101, USA; marisa.franklin512@topper.wku.edu; 3 Network for Hope, Louisville, KY 40223, USA

**Keywords:** organ donation, machine perfusion, ECMO, normothermic regional perfusion

## Abstract

The need for organs suitable for transplantation has continued to rise as need outweighs availability. Increased demand has driven innovation in the field. Over the past ten years, donation after circulatory death (DCD) donors have become a greater portion of the donor pool. This method of donation includes a period of warm ischemia time to the organs. Thus, its use is dependent on recovery methods. Historically, extracorporeal membrane oxygenation (ECMO) was one of the first pumping technologies to enhance organ preservation in the potential donor. Subsequently, the adoption of normothermic regional perfusion (NRP) technology has also shown promise in organ transplantation. These technologies have increased utilization of organs and enhanced the pool of donor organs. This review seeks to summarize the literature supporting in situ technologies (ECMO and NRP) utilized in procurement of solid organs from DCD donors. The benefit of in situ perfusion in DCD organ recovery is that these technologies increase the number of organs available for transplantation by reducing ischemic injury. The disadvantages include the added technical aspect, added operating room time, and the increased ethical concerns surrounding these technologies compared to conventional methods of organ recovery.

## 1. Introduction

The need for organs suitable for transplantation continues to rise worldwide. Thirteen people die each day in the United States waiting for a transplant [1]. Transplant systems worldwide have tried to address this discrepancy between availability and need. Strategies have varied by nation, yet many involve increasing the number of organs eligible for transplantation. Donation after circulatory death (DCD) has added greatly to the deceased donor pool. This type of donor is not new [2,3,4] but has been considered the way forward in increasing availability of organs for transplantation. While some ethical issues have been raised, this form of donation has become a major portion of deceased donors [5]. DCD includes a time interval with decreased organ perfusion (terminal phase) followed by a standard period of warm ischemia during the hands-off and recovery phases. As a result, there have been concerns surrounding the ischemic damage to organs procured from DCD donors. Reports of graft failure, dysfunction, rejection and ischemic cholangiopathy have impacted the utilization of DCD donors [6,7,8,9]. However, in 2023, DCD donors comprised 36% of the deceased donor pool in the United States [10]. Thus, many researchers have focused on how to best mitigate these known limitations for DCD donors through identifying “best practices” [11] and new technologies.

This narrative review will focus on the use of in situ machine perfusion technologies in organ transplantation. This review will highlight the advantages and disadvantages of these technologies. It will also discuss what has been shown in the literature and where more research is needed. The review was performed by searching PubMed for literature related to organ transplantation and in situ machine perfusion technologies in DCD donors. Search terms including “ECMO”, “NRP”, “DCD”, and “organ transplantation” were utilized. Studies related exclusively to ex vivo machine perfusion or donation after brain death (DBD) donors were excluded. Studies with cohorts < 10 were also excluded. Studies examining both DBD and DCD donors were not excluded. Reference lists of review articles obtained during literature search were also inspected for articles meeting inclusion criteria. This review will begin by outlining the current use of in situ machine perfusion in organ transplantation. A discussion of future directions of perfusion technology in organ transplantation will conclude the review.

## 2. ECMO

ECMO was developed to oxygenate blood in operations requiring prolonged cardiac bypass [12]. ECMO then developed into a therapy outside of the operating room. It was used to support patients with cardiac or respiratory failure where conventional support was inadequate [13]. Now, there are various implementations of ECMO in organ transplantation. ECMO can be used perioperatively for organ transplant recipients [14]. It can also be used to stabilize donors hemodynamically prior or during procurement [15]. Some receive extracorporeal cardiopulmonary resuscitation (ECPR) as a resuscitation strategy and are later identified to meet criteria for brain death [16,17].

Patients on ECMO can be considered for organ donation following brain death (DBD) or circulatory death (DCD) [18]. ECMO surrounding procurement is approached differently in these two classes of donors. There are several studies evaluating the use of ECMO in organ transplantation from DBD donors [19,20,21]. As this review focuses on DCD donors, we will discuss the literature assessing this population. However, research assessing DBD donors will be acknowledged in the case of studies that examined both DBD and DCD donors.

Fainberg et al. examined potential donors who were on ECMO at the time of referral to an organ procurement organization (OPO) in the United States [13]. Data was analyzed on 141 potential donors. Graft outcomes following transplantation were not included. This study found only 33% donated organs of the 141 potential donors on ECMO. Comparatively, 50% of non-ECMO patients donated organs. One may suspect this is due to a higher discard rate of organs. However, Fainberg et al. found a similar rate (11% to 10%) of organ discard between ECMO and non-ECMO donors. Amongst ECMO donors, the type of ECMO cannula (venovenous or venoarterial) was similar between those who donated and those who did not. This analysis includes both DBD and DCD donors who were on ECMO at time of OPO referral. They found that three of the five donors with discarded organs were from DBD donors and two were from DCD donors. This similar discard rate between DBD and DCD ECMO donors is promising. However, this study does not look at the outcomes of these transplanted organs, which is an important consideration when evaluating ECMO in donation.

Gregorini et al. evaluated eleven years of kidney transplant outcomes in DCD donors compared to ECMO prior to death (EPD) [22]. Their analysis showed that EPD can help reduce delayed graft function, post-transplant length of stay, complication rates, and need for dialysis. This study had a small cohort of 58 transplant recipients. Within this cohort, only 22 received organs from EPD donors. Importantly, although DCD donors were used as a comparison group, only 7 of 22 (32%) of kidneys from the EPD group were from DCD EPD donors. The other 15 kidneys (68%) were from DBD EPD donors. Thus, these findings may not be suggestive of all EPD DCD donors. Additionally, the EPD donor group had ECMO restarted to provide normothermic regional perfusion (NRP) for 1–4 h prior to procurement. Some kidneys in this cohort received additional hypothermic ex vivo machine perfusion. Thus, although this study has promising findings, further research is needed to characterize the effect of EPD in DCD donors for kidney transplantation.

Much of the research evaluating EPD in organ transplantation focuses on DBD donors. This is undoubtedly multifactorial. However, it is important to note there are ethical concerns with ECMO and DCD organ transplantation. These concerns could influence the lack of research in this population. As highlighted in this section, existing literature consists of a small cohort study with both DCD and DBD donors. Thus, more research is needed to truly evaluate the role of ECMO prior to death in DCD donation. After the declaration of death, ECMO technology, in the form of normothermic regional perfusion (NRP), has been utilized in the organ recovery process. Interest in this technology is rising rapidly. In the next section, we will discuss both forms of NRP and its role in DCD donation.

## 3. Abdominal Normothermic Regional Perfusion

**Figure 1:**
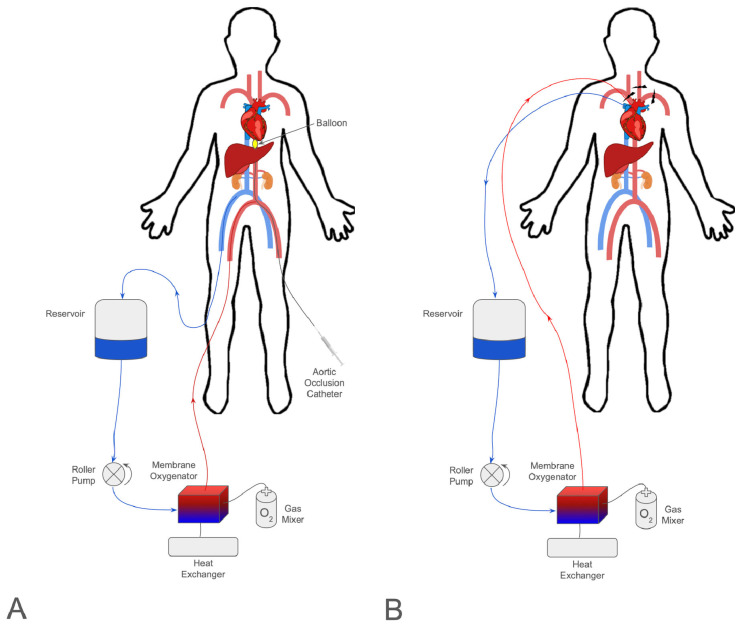
(**A**) Diagram of cannulation for normothermic regional perfusion (NRP). (**B**) Diagram of cannulation for thoracoabdominal normothermic regional perfusion (TA-NRP).

Normothermic regional perfusion (NRP) is implemented in organ donors during organ procurement. It is similar to ECMO with the primary difference being the timing of use. NRP is defined as ECMO used for the organ donor after the declaration of death. There are two types: NRP and thoracoabdominal normothermic regional perfusion (TA-NRP). The difference between NRP and TA-NRP rests on which organs are perfused [23]. In NRP, perfusion is restored to the abdominal organs. There is a clamp placed, or surgical ligation performed, at the level of the supraceliac aorta to prevent restoring perfusion to the brain (Figure 1A). In TA-NRP, perfusion is restored to the level of the aortic arch vessels allowing perfusion of the heart and lungs (Figure 1B). NRP provides organs with oxygenated blood to combat the effect of ischemia which can deplete ATP and produce oxygen free radicals. NRP’s use has increased in recent years, with an estimated 89% of organ procurement organizations (OPO) in the United States having some level of experience with NRP [24].

NRP has promising clinical evidence, summarized in Table 1. One such study was performed by Hessheimer et al. [25]. This group retrospectively evaluated outcomes of liver grafts procured from DCD NRP donors in Spain. This study examined 1165 livers considered for transplantation between 2012 and 2019. They compared the outcomes of recipients with grafts from NRP DCD donors to SRR DCD donors. Of note, high-risk recipients were not considered for super-rapid recovery (SRR). High risk was defined as recipients undergoing re-transplantation or with severely decompensated liver disease. This study found that NRP improved risk ratios of overall biliary complications, hepatic artery thrombosis, overall graft loss, and patient death. These results show promise for the use of NRP with DCD donors. However, it has limitations as a retrospective study where donors were not randomized between treatment arms.

Other researchers have examined the role of NRP in DCD liver procurement, but with smaller cohorts [26,27,30,38]. Bababekov et al., Bluhme et al., and Rodriguez et al. all showed comparable graft survival between DCD NRP and comparison groups. Comparison groups of these studies varied. Bababekov et al. compared NRP results to static cold storage [26], while Bluhme et al. and Rodriquez et al. compared results to DBD donors [27,30]. These studies showed non-inferiority, but no benefit to NRP. However, they did have smaller cohort sizes than the Hessheimer et al.’s study. Brubaker et al. conducted a study comparing NRP to super-rapid recovery (SRR) and found results similar to Hessheimer et al. [38]. Brubaker et al. found reduced biliary complications and early allograft dysfunction in the NRP group. These studies together suggest that NRP with DCD donors is superior to SCC or SRR with DCD donors. It is also implied that DCD grafts with NRP are non-inferior to DBD grafts. Yet many of these studies used specific DCD donor selection criteria during these comparisons. Thus, more research with larger cohorts and varied donor characteristics is needed to assert this claim.

Oniscu et al. performed a retrospective analysis of UK DCD donors between 2011 and 2019, where at least one abdominal organ was transplanted [29]. This study looked at utilization of abdominal organs (liver, kidney, pancreas) for transplantation and recipient outcomes. Organ-specific analyses were performed. However, for the pancreas-specific analysis, all NRP pancreas transplants were performed with simultaneous kidney transplant. Of note, although this study analyzed 4716 donors, only 163 underwent NRP. The small size of this cohort raises questions regarding generalizability. However, in this cohort, they found that NRP improved organ utilization rates. Specifically, NRP increased odds 3-fold for livers, 1.5-fold for kidneys, and 1.6-fold for pancreas grafts. Increased organ utilization was not the only benefit found in this study. NRP lowered the chance of developing delayed graft function in transplanted kidneys. NRP also showed a lower risk adjusted hazard ratio for graft failure in transplanted livers.

These studies display promising results for the use of NRP for DCD donors. It is important to note they are limited by their designs as retrospective analyses. However, with NRP and DCD donation it is difficult to design more rigorous randomized controlled trials. It is impossible to blind surgeons to the intervention arms. There is also significant hesitancy with randomization of interventions prior to inspection of the organs. However, if the randomization were conducted after inspection, it could be prone to selection bias. These factors influence the lack of RCTs to evaluate NRP and DCD donation. Although there are no randomized controlled trials, the existing evidence is positive. There are organs discarded worldwide from DCD donors due to concern for ischemic injury. NRP allows transplant surgeons to salvage some of these organs that might otherwise be discarded. Depending on the country and transplant allocation process, there is more time in the OR to reallocate organs that may have been initially turned down by the recovery surgeon but would be suitable for another recipient. Thus, NRP can increase the number of organs available for transplantation and decrease the time on an organ waitlist for patients.

## 4. Thoracoabdominal Normothermic Regional Perfusion

As discussed in the previous section, TA-NRP is an extension of NRP. TA-NRP includes the perfusion of thoracoabdominal organs. To perfuse the organs of the thoracic cavity, TA-NRP differs from NRP in its site of aortic occlusion (Figure 1B). Although these differing sites of aortic occlusion result in different ethical concerns, the science is largely the same. It can be used for thoracic and abdominal organs [37]. There have been a few small studies examining outcomes with TA-NRP in liver procurement [28,32]. In both studies, no patient developed ischemic cholangiopathy and only 4 patients of the total 43 (both cohorts) required dilation of a biliary stricture. Patients were followed for at least 1-year post-transplant. Survival was good, with only two patients succumbing to recurrent hepatocellular carcinoma. No primary non-function was observed and no patients required re-transplantation. The largest study to date reporting kidney outcomes after TA-NRP was reported by Zhou et al. [36]. They identified 306 donors recovered after TA-NRP, representing about 2% of all renal transplants from 2020 to 2022 in the UNOS dataset. Their analysis showed that TA-NRP renal donors were younger with lower KDPI than their matched cohorts. Delayed graft function was also lower but overall survival was similar when compared to direct-recovery donors. It is unsurprising that TA-NRP would have similar results to NRP for liver and renal transplantation, but these papers help confirm that assumption.

TA-NRP was specifically developed to increase the utilization and function of thoracic organs following a similar finding with NRP and abdominal organs. Kumar et al. evaluated the use of TA-NRP in DCD donors prior to heart procurement [33]. This study examined a small cohort of 32 patients who underwent transplantation from 2020 to 2023. Primary end point was 1-year survival, but secondary outcomes were also investigated. One-year survival in this cohort was 100%. There was one mortality, occurring 3 years after transplantation. Two patients in the cohort had primary graft dysfunction (PGD), with one requiring VA ECMO and one requiring an intra-aortic balloon pump. As this was an observational cohort, it involved analysis of a small population and there was no comparison to non-TA-NRP recipients.

In 2023, Siddiqi et al. published analyses of a larger cohort comparing outcomes between DBD and DCD heart allografts [34]. They examined outcomes at a single center for heart-only transplantations between 2020 and 2023. Survival, as well as secondary outcomes, was then compared between DBD and DCD donor grafts. Although this study did not specifically examine TA-NRP, 83% of the 122 DCD donors had received TA-NRP. There was no significant difference in survival between heart grafts from DBD or DCD donors. Increased utilization of DCD heart grafts could increase the donor pool significantly.

Aldrete et al. evaluated the use of TA-NRP in DCD donors for simultaneous heart and lung procurement [35]. The goal of this study was to assess if the utilization of lung grafts was affected by the increased use of hearts procured from TA-NRP DCD donors. In this cohort, lung procurement was higher amongst donors who had simultaneous cardiac procurement. TA-NRP was associated with improved survival and decreased 90-day mortality.

Transplantation of thoracic organs from DCD procurement is a growing area of transplantation. In the United States, the first heart transplant from a DCD donor occurred in 2019 [35]. Thus, in only a few short years the field has expanded dramatically. As this is a new technology, there is room for additional research guiding its use. However, the existing studies appear promising. TA-NRP can increase the number of heart and lung grafts available for transplantation. Yet there remain ethical concerns over TA-NRP. The UK and Spain have moved away from the use of TA-NRP with clamping the supra-aortic arch vessels due to concerns over its inadequate safety [39]. However, TA-NRP is not alone in this regard. Some USA OPOs report that many hospitals within their jurisdiction have banned the use of both TA-NRP and NRP over ethical concerns [24]. Therefore, despite the promising clinical evidence outlined in this review, the future of NRP in DCD donation remains in question as further data is collected on its safety and use.

## 5. Ethical Concerns Surrounding ECMO, NRP, and TA-NRP in DCD Donation

Ethicists have raised concerns with the use of in situ perfusion technologies in DCD donors. Some argue it invalidates the previous declaration of death (dead donor rule). The dead donor rule is a fundamental principle of ethics in organ transplantation. Its challenge is a serious area of concern. Additionally, there are questions of the brain being re-perfused in DCD donors [40]. This issue is irrelevant in the case of DBD donors due their lack of neurologic function. However, in DCD donors, neurologic function can vary at time of procurement.

When examining the impact of in situ perfusion on the dead donor rule, it is important to understand its principle. The dead donor rule (DDR) states that (1) organ donors must be declared dead prior to organ procurement and (2) organ donors do not die because of organ procurement. In situ perfusion, including ECMO, NRP, and TA-NRP, is used after patients are declared dead. The argument, for those opposed to in situ perfusion in DCD, is the determination of death is on circulatory grounds. In situ perfusion for organ donation could be considered to “resuscitate” the donor by restoring circulation. Thus, the donor no longer meets criteria for circulatory death, “negating the determination of death”. Then, if the procurement were to proceed, it would be the procurement itself that results in the death of the donor.

Although it can be easy to understand the ethical concerns with this technology, there are conflicting viewpoints among experts [41]. One objection is that many organ donors have consented for donation. It is sometimes the final wish of the donor and their family to help others through organ donation. Thus, failing to allow these donors to participate could violate their autonomy [42]. Others argue it cannot be known for sure if restoring circulation truly “resuscitates the donor”. Anoxia causes damage to the brain. The brain anoxia associated with circulatory death will ultimately lead to neurologic death. However, it is not known over what time frame this occurs or if it varies. If there is concern with the length of no-touch wait time, an alternative could be lengthening the no-touch wait time in the case of DCD donation when in situ perfusion is used. However, this change should not occur based on intuition alone. If there were data to support a certain threshold for the irreversibility of circulatory death, this would be valuable in this debate. There are some ethicists who have argued whether the reversibility is even of moral importance [42]. However, this is not the view held by most clinicians.

In the case of in situ perfusion, especially TA-NRP, perfusion to the aortic arch raises concerns of restoring perfusion to the brain during organ procurement. Surgeons attempt to prevent this through varied means such as aortic occlusion, carotid ligation, or venting of arch vessels. Yet ethicists have questioned whether this is sufficient. However, this is a question best answered with data. Scientists can test to see if brain perfusion occurs after NRP or TA-NRP initiation despite aortic occlusion during procurement. If it were proven that brain perfusion was restored despite aortic or neck vessel occlusion, then NRP or TA-NRP should not be used in DCD donors. Restoring perfusion to the brain in a donor with neurologic function undergoing organ procurement would be ethically impermissible. This is the reason ligation/occlusion maneuvers are performed to prevent restoring circulation to brain. However, this concern of perfusion despite ligation/occlusion maneuvers has only been hypothetical.

Frontera et al. first reported in 2023 a small case series utilizing transcranial Doppler studies during TA-NRP to confirm no cerebral blood flow following ligation of the brachiocephalic, left carotid and left subclavian arteries [43]. As a control group, the same technology was performed on patients undergoing cardiothoracic surgery while on ECMO and Impella mechanical circulatory support. No flow was observed with the TA-NRP patients, while normal cerebral flow was maintained during surgery on the ECMO patient. Another small series of 10 patients undergoing either NRP or TA-NRP measured intracranial pressure in the circle of Willis using invasive means [44]. There was no significant pressure measured after aortic occlusion in either group of patients, with the conclusion being that cerebral perfusion does not occur during NRP. The same group with Royo-Villanova et al. assessed brain perfusion using a radiotracer and perfusion scintigraphy during NRP and TA-NRP [45], the same scintigraphy utilized in brain death declaration to confirm no cerebral blood flow. In this study, they found no radiotracer uptake in the brain after aortic balloon occlusion, data that is reassuring for the continued growth of TA-NRP and NRP. As these three studies use varied methods to measure cerebral perfusion, their shared conclusions suggest that brain reperfusion is not observed in NRP.

The focus of this review is not on ethical issues alone. However, it is difficult to discuss these technologies without acknowledging their surrounding debates. Ethical concerns are not the only disadvantage of in situ perfusion technology. ECMO and NRP can add technical aspects to the recovery process alongside increased operating room time. However, these are thought to be outweighed by the benefit of increased organ grafts for transplantation. Yet ethical concerns cannot be addressed as easily. Although increasing the organs available for transplantation is important, it is necessary to only engage in what is ethically permissible. Although the current literature supports the use of in situ perfusion in DCD donation, there remain debates amongst ethical experts. These debates impact the scientific literature on the topic. Ethical concerns have resulted in hesitancy in the use of in situ perfusion technologies in DCD donors, resulting in less research to evaluate its use. The resolution of this debate will influence the future of in situ perfusion in DCD donors.

## 6. Future Directions

Although this review focuses on in situ perfusion technologies, future advances in the field will likely include a combination of in situ and ex vivo perfusion. Broadly, ex vivo perfusion refers to machine perfusion of organs after they have been removed from an organ donor. There are various methodologies and perfusion technologies available within the field of ex vivo perfusion. A thorough discussion of these technologies is outside the scope of this review article. However, it is important to acknowledge that the future of in situ perfusion will likely grow in tandem with ex vivo perfusion. Investigators have already begun to examine how these technologies can be used together. Croome et al. evaluated NRP livers followed by a period of ex vivo normothermic machine perfusion [46]. They observed lower rates of ischemic cholangiopathy with improved graft survival with NRP alone or NRP + NMP compared with cold storage from DCD donors. Specific differences in outcome between NRP alone and NRP + NMP were not significant. However, NRP + NMP allowed for longer transport times and complicated recipient management. Conversely, Patrono et al. assessed the sequential addition of oxygenated hypothermic machine perfusion with NRP [47]. They found that the incidence of anastomotic biliary complications and ischemic cholangiopathy was not different than propensity-matched brain-dead donors, nor was one-year patient and graft survival. This study concluded that the combination of oxygenated hypothermic machine perfusion with NRP could achieve outcome rates similar to those of DBDs. Clinical practice guidelines have not been developed for the combination of modalities (NRP + MP), but many centers are rapidly adopting this practice. Survey data in 11 of the 21 transplant centers in Italy showed that nearly all were using NRP for DCD donors followed by a period of machine perfusion, whether hypothermic or normothermic [48]. However, additional research is needed to determine the optimum implementation of NRP and machine perfusion in DCD donors.

## 7. Conclusions

This review highlighted the literature surrounding in situ perfusion technologies in DCD organ procurement. The advantage of in situ perfusion in DCD organ recovery is that it increases the number of organs available for transplantation by reducing ischemic injury. The disadvantages include the added technical aspect, added operating room time, and the increased ethical concerns surrounding these technologies compared to conventional methods of organ recovery. Although ECMO will continue to be used prior to death declaration, NRP will likely play a larger role in increasing organs for transplant in DCD donation. The literature included in this review is promising for the future of in situ perfusion in DCD donation. However, it is important to acknowledge that as these are new technologies, implementation methods have been diverse between studies. As more research focuses on this topic, it will become clearer how to standardize the utilization of these technologies. Very little data exists using a combination of these modalities (ECMO/NRP with ex vivo machine perfusion). Yet their combined use could increase options available for transplant surgeons. Increasing options could lead to further increases in organs suitable for transplantation. Additionally, with increased storage times and transportation while on machine perfusion, a broader sharing of organs geographically will be available. The recent development of organ rehabilitation centers and centralized organ recovery centers suggests that these technologies will be a part of achieving the goal of increased organs for transplantation and decreased deaths on a transplant waiting list [49,50,51].

## Figures and Tables

**Table 1:** Summary of the literature on NRP and TA-NRP outcomes.

Technique	Organ	*n*	Outcomes	References
NRP	Liver	*n* = 162 NRP = 97 SCS = 79	Comparable 12 mo allograft and recipient survival for NRP and SCS, despite higher risk donor–recipient pairing in NRP group.	Bababekov et al. [26]
		DCD NRP = 18 DBD = 28	Comparable recipient and graft survival, early allograft dysfunction, and AKI between DCD NRP and DBD groups.	Bluhme et al. [27]
		*n* = 242 NRP = 106 SRR = 136	Reduced rates of ischemic cholangiopathy, biliary complications, and early allograft dysfunction in NRP group.	Brubaker et al. [28]
		*n* = 1165 NRP = 775 SRR = 390	NRP cohort showed reduced rates of complications	Hessheimer et al. [25]
		N_total_ = 4716 Non-NRP = 4553 NRP = 163	NRP increased odds of liver being transplanted 3-fold. Improved 1-year survival	Oniscu et al. [29]
		*n* = 117 DCD NRP = 39 DBD = 78	Comparable survival, early allograft dysfunction and primary non-function between NRP and DBD groups.	Rodriguez et al. [30]
	Kidney	N_total_ = 4716 Non-NRP = 4553 NRP = 163	NRP increased odds of kidney being transplanted by 1.5-fold; 35% lower chance of DGF in NRP cohort	Oniscu et al. [29]
		*n* = 632 DCD = 229 NRP = 29	Delayed graft function rate lower in NRP group. One-year graft loss rate was lower in NRP group.	Pearson, et al. [31]
	Pancreas	N_total_ = 4716 Non-NRP = 4553 NRP = 163	NRP increased odds of pancreas being transplanted 1.6-fold	Oniscu et al. [29]
TA-NRP	Liver	*n* = 43	Good early allograft and recipient outcomes in small cohort.	Brubaker et al. [28]
		*n* = 13	Observation study with small cohort; 92% of cohort alive with good liver function at median follow-up of 439 days.	Sellers, et al. [32]
	Heart	*n* = 32	Observational study. Cohort had 100% 1-year survival.	Kumar et al. [33]
		*n* = 385 DCD = 122	DCD heart graft outcomes were non-inferior to DBD outcomes.	Siddiqi et al. [34]
	Lungs	N_tot_ = 24,431 TA-NRP_cardiac_ = 325	Improved 90-day and overall survival.	Alderete et al. [35]
	Kidney	N_tot_ = 16,140 TA-NRP = 306	Similar survival and all-cause graft failure between TA-NRP and direct-recovery groups. Lower likelihood of delayed graft function with TA-NRP.	Zhou et al. [36]
	Multi	TA-NRP = 22	Comparable recipient and graft survival between TA-NRP group and DBD group	Motter et al. [37]

Legend: NRP = normothermic regional perfusion; TA-NRP = thoracoabdominal normothermic regional perfusion; DGF = delayed graft function; SRR = super-rapid recovery, SCS = static cold storage.
